# Circulating cell-free DNA, telomere length and bilirubin in the Vienna Active Ageing Study: exploratory analysis of a randomized, controlled trial

**DOI:** 10.1038/srep38084

**Published:** 2016-12-01

**Authors:** Anela Tosevska, Bernhard Franzke, Marlene Hofmann, Immina Vierheilig, Barbara Schober-Halper, Stefan Oesen, Oliver Neubauer, Barbara Wessner, Karl-Heinz Wagner

**Affiliations:** 1 Research Platform Active Ageing, University of Vienna, Althanstrasse 14, 1090, Vienna, Austria; 2 Centre for Sport Science and University Sports, Department of Sport and Exercise Physiology, University of Vienna, Auf der Schmelz 6, 1150 Vienna, Austria; 3 Department of Nutritional Sciences, University of Vienna, Althanstrasse 14, 1090, Vienna, Austria; 4 Queensland University of Technology, Faculty of Health, School of Biomedical Sciences, Institute of Health and Biomedical Innovation (IHBI), Tissue Repair and Regeneration Group, 60 Musk Avenue, Kelvin Grove Campus, Brisbane, QLD 4059, Australia

## Abstract

Telomere length (TL) in blood cells is widely used in human studies as a molecular marker of ageing. Circulating cell-free DNA (cfDNA) as well as unconjugated bilirubin (UCB) are dynamic blood constituents whose involvement in age-associated diseases is largely unexplored. To our knowledge, there are no published studies integrating all three parameters, especially in individuals of advanced age. Here we present a secondary analysis from the Vienna Active Aging Study (VAAS), a randomized controlled intervention trial in institutionalized elderly individuals (n = 101). Using an exploratory approach we combine three blood-based molecular markers (TL, UCB and cfDNA) with a range of primary and secondary outcomes from the intervention. We further look at the changes occurring in these parameters after 6-month resistance exercise training with or without supplementation. A correlation between UCB and TL was evident at baseline (p < 0.05), and both were associated with increased chromosomal anomalies such as nucleoplasmatic bridges and nuclear buds (p < 0.05). Of the three main markers explored in this paper, only cfDNA decreased significantly (p < 0.05) after 6-month training and dietary intervention. No clear relationship could be established between cfDNA and either UCB or TL. The trial was registered at ClinicalTrials.gov (NCT01775111).

Ageing is accompanied by an accumulation of macromolecular damage, leading to cellular dysfunction and ultimately, tissue and organ malfunction^1^. In humans the ageing process appears to be a mixture of stochastic events leading to a high variability of phenotypical outcomes between individuals of the same age.

Telomere length is one of the most commonly used markers of ageing^2^. Telomere shortening is a result of the mitotic activity of a cell, and in humans it roughly corresponds to the age of the organism^3^. On the other hand, telomere length has a limited power as an age biomarker due to a very high inter-individual variation^4^,^5^. While a clear decline is visible when comparing healthy individuals throughout a broad age range, this difference is often negligible over a more narrow range. This is especially the case when comparing individuals at a more advanced age, when telomeres appear to shorten at a reduced rate^6^. Moreover, studies in humans often use blood cells as a test material for measuring telomere length^7^,^8^. As blood cells comprise a mixture of different individual cell types with different mitotic history, telomere length measured in these samples can additionally show a large intra-individual variability^7^,^9^. Therefore, there is an increased need for identifying reliable and clinically accessible molecular markers of ageing, which would complement telomere length as a marker^10^.

It has been previously shown^11^,^12^,^13^ that chromosomal abnormalities arise more frequently in the elderly population. The increased chromosomal instability, in absence of malignancy, will ultimately lead to increase in cell death. Dying and dead cells release their content extracellularly, and a portion of the genomic DNA can be found circulating in the bloodstream before final excretion as a waste product^14^. A recent review^15^ suggests the possibility of cell-free genomic-derived DNA (cfDNA) integration into the genome of living cells, leading further to increased genome instability.

Breitbach *et al*.^16^ proposed that this circulating cfDNA can be additionally excreted in an active manner from living cells and could play a role in cell signalling. Circulating concentrations of cfDNA have been shown to increase rapidly after strenuous exercise, and return to normal within hours or days^16^. However, much of the physiological and pathophysiological relevance of cfDNA remains unknown, so far.

Jylhava *et al*.^17^,^18^,^19^ have investigated cfDNA as a possible ageing biomarker in healthy individuals. In a genome-wide association study (GWAS) a potential involvement of the UDP-glucuronosyltransferase UGT1A1 in the excretion of cfDNA was revealed^20^. This enzyme is highly specific for metabolising unconjugated bilirubin (UCB) into water-soluble products that can be easily excreted from the organism^21^. However, there is no published data looking at a potential relationship or interaction between UCB and cfDNA in humans.

Unconjugated bilirubin has been suggested to play an important role in human disease prevention, especially in relation to age-associated diseases^22^. A number of epidemiological studies have found an inverse relationship between increased serum bilirubin and age-associated disease incidence^23^,^24^,^25^. We have recently identified a relationship between mildly increased UCB, characteristic for a condition called Gilbert’s Syndrome, and telomere length^26^. These individuals present with serum bilirubin levels above 17.1 umol/L whereas non-affected individuals range between 3 and 17.1 umol/L. There are indications that even in non-Gilbert’s Syndrome individuals concentrations higher than 10 umol/L can be indicative for a reduced disease risk^23^. However, it is not clear whether this intermediately increased UCB concentration is linked to longer telomeres in non-affected individuals.

The Vienna Active Ageing Study (VAAS) has been investigating different aspects of the ageing process in institutionalized elderly individuals, and the effects of resistance training and nutritional supplementation intervention on overall fitness and health-related parameters. Published data on this study have shown an improvement in physical performance in individuals undergoing an exercise training intervention, compared to sedentary controls^27^, as well as a trend for a decreased chromosomal and DNA damage in the entire study population^28^. The main aim of this paper was to investigate a potential relationship between blood telomere length, cfDNA and unconjugated bilirubin at baseline and after a 6-month training intervention. We further conducted an exploratory analysis in order to identify relationships between cfDNA, UCB and TL on one side and chromosomal damage endpoints, physical performance measures, CVD markers and oxidative damage end products on the other. Based on previous knowledge we hypothesized that there is an inverse relationship between TL and UCB on one side, and cfDNA and markers of chromosomal instability on the other.

## Results

### Baseline characteristics of the entire cohort

The baseline study characteristics have been described elsewhere^11^. From the 105 individuals that complied to the initially established inclusion criteria^15^,^27^, we additionally excluded 4 individuals with increased reference values of liver enzymes (ALT, AST or GGT). Increased liver enzymes were important indicators for liver abnormality which might influence bilirubin levels, a key variable in this analysis. As the percentage of males in this study population was too low to achieve statistical relevance, we carried out all further analyses on a pooled population of both genders. Supplementary Figure S1 shows that no clear distinction and clustering can be seen between males and females using a Principal Component Analysis (PCA).

Five individuals fitted the criterion for Gilbert’s Syndrome (UCB concentration ≥17.1 μM), corresponding to an incidence of about 5% in our study population. This goes in line with the estimated incidence of this condition in the general population^29^. PCA analyses showed that these individuals cluster close together, but are not distinct from the rest of the participants in this study cohort (Supplementary Figure S2).

### Correlations at baseline

**Figure 1:**
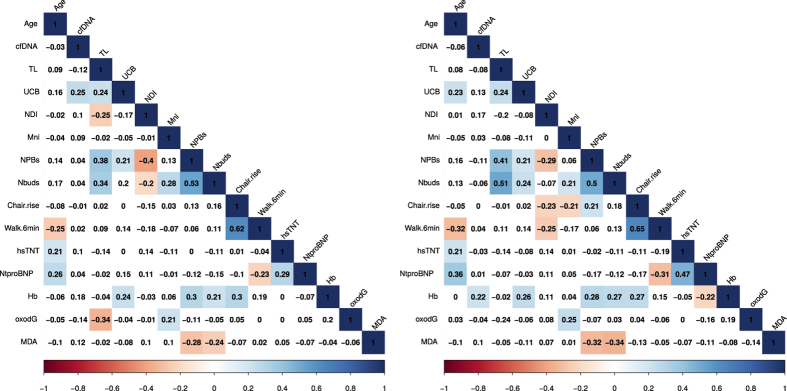
Pearson and Spearman correlation matrices at baseline. Values of Pearson r or Spearman ρ coefficients are given in the correspondent glyph. Pearson correlation matrix (left panel); Spearman correlation matrix (right panel). The glyphs are marked with colour only if they show a correlation at P < 0.05 level. Blue colour represents positive correlation whereas red represents negative correlation. Numerical p-values are shown in Supplementary Table S1. Abbreviations and units are given in the supplementary material.

We used Pearson and Spearman correlation matrices (Fig. 1) to explore collinearity between variables and baseline. As most variables were non-parametric (Supplementary Figure S3.) we only considered Spearman statistics (Supplementary Table S1). Pearson correlation matrix is shown only as a descriptive. We summarize only the most important observations.

Age had no effect on cfDNA levels in the study population at baseline. Additionally, we could not detect any relationship between age and telomere length. UCB, on the other hand, showed a monotonic increase with age in our study sample (ρ = 0.23, p = 0.025).

Although baseline UCB showed a moderate positive Pearson correlation with cfDNA (r = 0.257), Spearman statistics showed no significance. In contrast, telomere length and UCB showed a significant correlation at baseline (ρ = 0.238, p = 0.047). In addition, a relationship of UCB with blood haemoglobin was observed (ρ = 0.26, p = 0.012).

Both UCB and telomere length at baseline correlated positively with the number of nucleoplasmatic bridges (NPBs) (ρ = 0.21, p = 0.043 and ρ = 0.41, p = 0.001, respectively) and nuclear buds (NBuds) (ρ = 0.24, p = 0.019 and ρ = 0.51, p = 0.004). On the contrary, MDA showed an inverse correlation with both NPBs and NBuds (ρ = −0.32, p = 0.001 and ρ = −0.34, p < 0.001), but not with TL or UCB. Whole blood telomere length and urinary levels of oxidized guanine showed a negative Pearson correlation (r = −0.34) which was not significant using Spearman statistics. There were no significant correlations between TL and cfDNA and any physical performance parameters such as chair rise test or 6-minutes walking test.

### Six-months-finishers at baseline

The study participants were randomly assigned to three intervention groups: resistance exercise training (RT), resistance training and supplementation (RTS), and a control group (CT) undergoing a cognitive intervention only. Table 1 shows the baseline characteristics of the 6-months finishers, divided by intervention groups.

### Changes after six months exercise training

**Figure 2:**
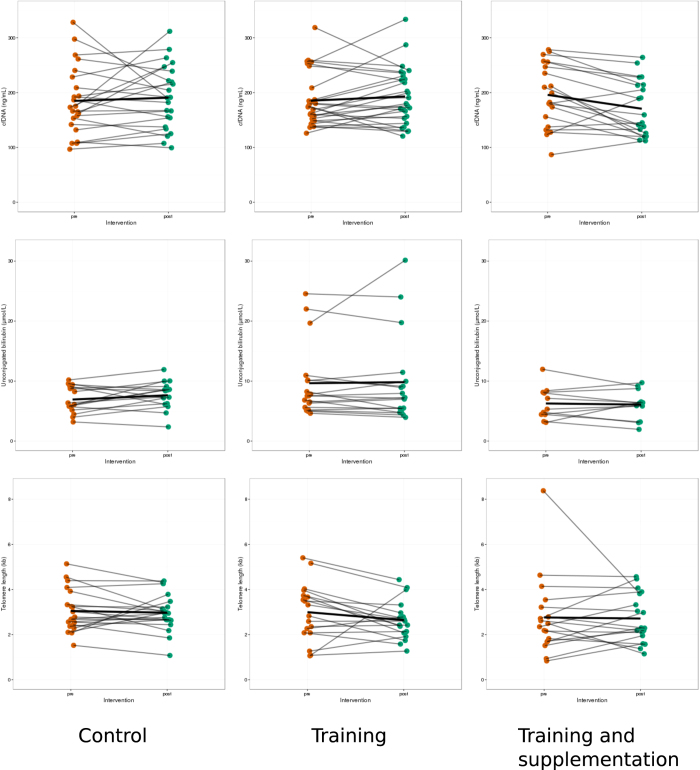
Paired-differences between pre-and post-intervention values for the main parameters. Each orange dot represent a value pre-intervention and green dots are 6-months post-intervention. Bold black line represents the average change. Only cell-free DNA in the RTS group exhibited a significant decrease (p = 0.009, paired t-test). Control group, number of pairs included: cfDNA, n = 23; TL, n = 20; UCB, n = 16. Training group, number of pairs included: cfDNA, n = 27; TL, n = 18; UCB, n = 18. Training and supplementation group, number of pairs included: cfDNA, n = 20; TL, n = 18; UCB, n = 11.

Cell-free DNA concentrations were reduced significantly after 6 months RTS intervention (t-statistics = 2.92, p-value = 0.008), but not in the CT or RT-only groups. There was no significant difference in telomere length or UCB concentrations in any of the intervention groups after 6 months, as shown is Fig. 2.

**Figure 3:**
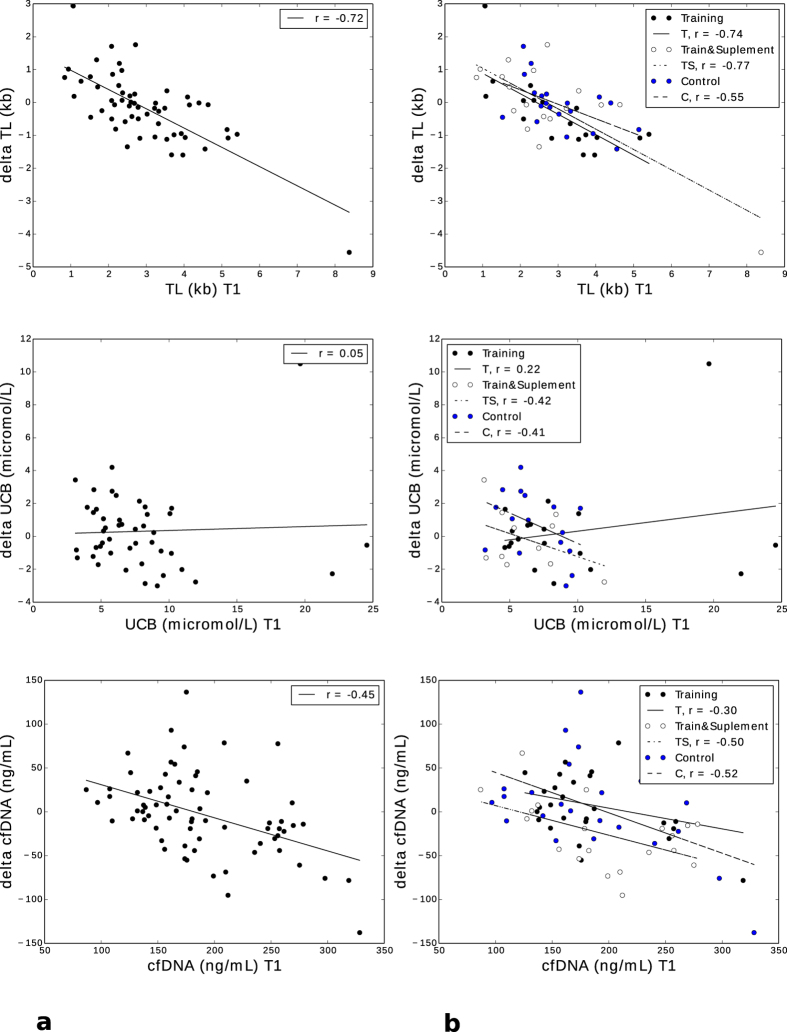
Scatter plot depicting correlations in the main variables at baseline to the respective 6-month change: (**a**) entire study population (TL, p < 0.001; UCB, non-significant; cfDNA, p < 0.001). (**b**) Divided by intervention group (TL: RT and RTS group: p < 0.001, CT group: p = 0.011; UCB: no group reached significance; cfDNA: RT group n.s., RTS: p = 0.025, CT: p = 0.012).

We consecutively determined whether the baseline concentrations of our key variables correlated with the change in the same parameter after 6 months intervention. The 6-month change in telomere length correlated inversely with telomere length at baseline in the entire cohort and all intervention groups separately (p < 0.001, Fig. 3a). The same was observed for cfDNA concentrations in the entire cohort (p < 0.001, Fig. 3b), even though the correlation did not reach significance in the RT group. UCB concentrations at baseline had no influence on the observed 6-month change in any of the intervention groups (Fig. 3c).

### Correlations after six months exercise training

**Figure 4:**
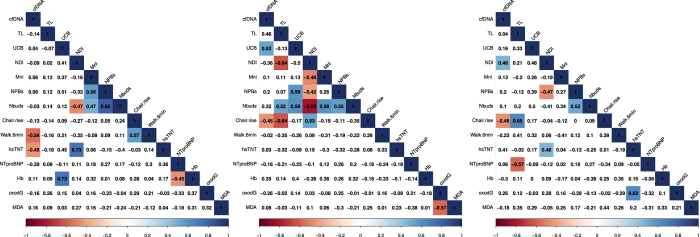
Spearman correlation matrices describing relationships between changes after 6-month intervention training, stratified by intervention group. Values of Spearman ρ coefficients are given in the correspondent glyph. The glyphs are marked with colour only if they show a correlation at P < 0.05 level. Blue colour represents positive correlation whereas red represents negative correlation. Control group CT, n = 9–22 (left panel); resistance training and supplementation group RTS n = 9–22 (middle); resistance training group RT, n = 10–29 (right panel). Numerical P-values are shown in Supplementary Table S2. Abbreviations and units are given in the supplementary material.

We next aimed to investigate which parameters exhibit a similar pattern with regards to negative or positive change after 6 months intervention, using a Spearman correlation matrix. The correlation matrices stratified by intervention groups are depicted in Fig. 4. We briefly describe the most relevant correlations.

Changes in telomere length and cfDNA did not correlate to each other in any of the intervention groups. An increase in cfDNA was associated with decreased chair rise repetitions in the CT and RTS groups, whereas a deterioration of the 6 min walking test was accompanied by an increase in cfDNA in the RT group only. Longer telomeres were associated with an improvement in the chair rise test in the RTS group but with a decrease in the TS group (ρ = 0.65, p = 0.005 and ρ = −0.64, p = 0.01).

An increase in UCB in the CT group correlated strongly with the increase in blood hemoglobin (ρ = 0.73, p = 0.002). In the RT group, UCB concentration increase correlated with an increase in both NPBs (ρ = 0.59, p = 0.02) and NBuds (ρ = 0.59, p = 0.019). In the same group telomere length shortening was accompanied by an increase in intracellular chromosomal damage, such as formation of NBuds (ρ = 0.52, p = 0.037) and a decrease in nuclear division index (ρ = −0.64, p = 0.007).

A positive correlation between the observed changes in UCB and cfDNA was evident in the RT group only (r = 0.538, p = 0.032). An increase in UCB was correlated with an increase in DNA damage parameters, especially NPBs, in both training groups (RT group: r = −0.576, p = 0.024; RTS group: r = −0.611, p = 0.060).

## Discussion

The present study investigated the relationship between circulating cell-free DNA, serum unconjugated bilirubin and telomere length in a predominantly female elderly population. To our knowledge, this is the first study investigating the interplay between these parameters within elderly individuals. Moreover, the novelty of these data is in linking these markers in response to a prolonged intervention involving exercise training with and without a dietary supplementation.

Telomere length and circulating cell-free DNA at baseline were not affected by age in this study population. The possible reason for this finding is the advanced age of the study participants leading to a relatively broad variance in most baseline parameters. All study participants were between 65 and 98 years of age, where the discrepancy between biological and chronological age is the greatest. Additionally, it is very plausible that individuals that have reached a more advanced age in good health have a more favourable molecular ageing pattern than their slightly younger peers^30^.

We hypothesized that telomere length at baseline would be inversely associated with markers of chromosomal instability and oxidative DNA damage. In contrast, we expected cell-free DNA to increase with the levels of chromosomal damage. Contrary to our hypothesis, longer telomeres were associated with increased chromosomal instability at baseline, measured by an increase in nucleoplasmatic bridges (NPBs) and nuclear buds (NBuds). Nucleoplasmatic bridges are formed as a result of erroneous DNA repair and telomere fusion of two chromosomes^13^. This process is triggered by critical telomere shortening and uncapping. However, to our knowledge, there is no indication that NPBs formation can lead to telomere elongation. Some evidence suggests that in a case of folate deprivation NBuds might contain a larger amount of telomeric DNA, leading to an abnormal increase in telomeric content^31^. The present study participants indeed had an inadequate baseline level of folate^28^ in erythrocytes, which might partly explain our results.

Baseline concentrations of cfDNA did not reflect the extent of DNA or chromosomal damage in PBMCs occurring simultaneously, measured by the CBMN assay end-points such as micronuclei frequencies, NPBs and NBuds formation. Notably, cfDNA levels in plasma did not appear abnormally high for any of the study participants. For cfDNA integration into healthy cells^16^, it is likely that much higher levels of cfDNA would be required.

There was a weak positive correlation between telomere length and UCB at baseline. This is in agreement with our recent results suggesting an occurrence of longer telomeres in individuals with Gilbert’s Syndrome, a condition of mild chronic hyperbilirubinaemia^26^. Approximately 5% of our study population matched the criterion for GS diagnosis, showing increased UCB levels over the diagnostic minimum for GS. Interestingly none of the potential GS showed a tendency for short telomeres (Supplementary Figure 4a). However, it this setting, the group size was too small to make a statistically sound conclusion. It should be taken in consideration that the present study was conducted using a different starting material for DNA extraction (i.e. whole blood instead of isolated PBMCs) which excludes the possibility for direct comparison between the studies.

The relationship between UCB levels and cfDNA appears to be more complex. Even though there was no direct significant relationship between the two, the levels of cfDNA were consistently higher in individuals suspected for GS. These individuals have distinctly higher UCB levels and appear as outliers to the entire group (Supplementary Figure 4b). As we have no information on the UGT1A1 genotype of participants in the present study, we could only assume that the ones showing an increased UCB concentration indeed carry a mutation in their UGT1A1 gene which additionally slows down the excretion of circulating cfDNA entities. Another possibility is that the free UCB fraction in plasma is binding to the free DNA fraction. A study has reported that bilirubin can bind DNA in presence of Cu(II)-ions, which could promote oxidative DNA damage^32^. However, more research is needed in order to get an insight if UCB and cfDNA are directly interacting, or are merely targets of the same metabolic pathway.

The main outcomes of the Vienna Active Ageing Study related to improvement in physical fitness parameters have been described earlier^27^. Briefly, functional parameters have been found to improve over time and related to the training intervention. In addition, chromosomal damage, such as micronuclei frequencies or NPBs showed a trend to decrease in all three groups^28^. The supplementation group acquired improvements in plasma vitamin B12 and folate status in erythrocytes^28^. While acute exercise increases the concentration of cfDNA within hours post-exercise, it is being quickly eliminated and returns to normal values within a few days^16^. However, the effect of regular moderate exercise on long-term cfDNA levels has not been reported so far. We observed a significant decrease in cfDNA levels in the RTS group after 6 months. There was no association between cfDNA and B12 or folate or the changes occurring in these parameters between the two time-points (data not shown). It is possible that there was an indirect effect of the latter two on the reduced cfDNA in plasma. As the role of circulating cfDNA is not yet well defined, we cannot exclude that the observed reduction in the training and supplementation group might not be beneficial at all as cfDNA could potentially play a role in the training adaptation.

Mean telomere length has not changed drastically throughout the course of 6 months. It has been shown previously that telomeres shorten at a rate of only about 30 bp/year which is difficult to detect with any standard telomere measurement method^33^. However, there have been reports^7^ of telomere length fluctuations with time in whole blood samples, which occurs due to a shift in cell populations throughout the intervention period. Similar to the observations from *Svenson et* *al*., there was an inverse correlation between telomere length measured at baseline and the 6-month change, independently from the training intervention. These intra-individual fluctuations in blood telomere length should be considered carefully when using telomere length as a marker, which is its main limitation.

Similarly to the outcomes at baseline, the change in measured intracellular damage parameters in the training group is closely followed by a change in TL and UCB. Additionally, and contrary to our expectations, telomere shortening correlated with higher improvement in the chair rise test. It is unclear from our results if there is any causal relationship between these parameters.

Taken together, we have shown a relationship between telomere length, unconjugated bilirubin and chromosomal anomalies in an elderly population, in the light of a lifestyle intervention. On the other hand, cfDNA showed no connections to any of the tested parameters. Perhaps, due to the very transient nature of cfDNA, a study dealing with acute outcomes would be more appropriate. Instead, we can emphasize the potential role of UCB as a key player in age-associated cellular signalling.

## Materials and Methods

### Subject recruitment and sample preparation

A total of 117 (of which blood samples were available from 105) institutionalized elderly individuals aged 65–98 years (both men and women) were recruited from five different senior residencies in Vienna, Austria (Curatorship of Viennese retirement homes)^11^,^27^. The subjects were mentally (Mini Mental State Examination ≥23) and physically (Short Physical Performance Battery >4) able to participate in the study. Furthermore, they were sedentary (<1 h of physical activity or exercise per week) and free of conditions contraindicated with medical training therapy or measurement of physical performance, such as cardiovascular diseases, diabetic retinopathy and regular use of cortisone-containing drugs. Regular strength training (>1×/week) in the last 6 months before the study beginning was an exclusion criterion. Written informed consent was obtained from all participants before entry, and the study was performed in accordance with the Declaration of Helsinki. This study was approved by the ethics committee of the City of Vienna (EK-11-151-0811) and registered at ClinicalTrials.gov (NCT01775111) on January 21^st^, 2013.

The study was conducted in a randomized, controlled, observer-blind design. The participants were randomly divided into three groups – cognitive training (CT), resistance training (RT), RT + protein supplement (RTS) – and matched for gender. Blood samples were taken and physical performance tests were executed before (T1 or pre), and after six months (T3 or post) of intervention.

Plasma values of liver enzymes (AST, ALT, GGT) elevated over 10% above the reference values was an additional exclusion criterion, as unconjugated bilirubin can be affected by altered liver function. This lead to exclusion of additional 4 study participants.

Blood samples were collected early morning after an overnight fast using heparin, serum and EDTA tubes (Greiner Bio-One, Kremsmunster, Austria). Peripheral blood lymphocytes were isolated using Ficoll separation tubes (Greiner Bio-One).

### Study intervention design

A detailed description on the training protocol, protein and micronutrient supplementation and cognitive training are given by Franzke *et al*.^28^. Briefly, the resistance training groups (RT and RTS) received two weekly sessions of resistance training, conducted on two non-consecutive days and supervised by a sport scientist. Exercises were conducted using elastic bands, chairs and own body weight^27^. In the initial phase (4 weeks) one set of 15 repetitions was performed in order to learn the correct form of each exercise. From the fifth week on the intensity and volume has progressively been increased from two sets of light exercises to two sets of heavy resistance. If the participants could easily perform two sets of 15 repetitions they were told to either take more resistance or to perform a more difficult version of the exercise.

The RTS group additionally received a nutritional supplement every morning, as well as directly after each training session. Each drink supplied a total energy of 150 kcal and contained 20.7 g protein (56 energy (En)%, 19.7 g whey protein, 3.0 g leucine, >10 g essential amino acids), 9.3 g carbohydrates (25 En%, 0.8 BE), 3.0 g fat (18 En%), 1.2 g roughage (2 En%), 800IU (20 μg) of vitamin D, 250 mg calcium, vitamins C, E, B6 and B12, folic acid and magnesium (FortiFit, NUTRICIA GmbH, Vienna, Austria).

The CT group performed coordinative or cognitive tasks two times per week, equally to the RT and RTS groups in order to avoid socialization bias. Participants of all groups were instructed to maintain their regular food intake.

### Chair rise test

To perform well in the chair rise test, the participants had to stand up from a chair (46 cm seat height) as often as possible within 30 s. To ensure a safe test-setting, the chair was placed against the wall. For one successful repetition, participants had to fully stand up (hip and knee fully extended) and sit back, with their arms crossed over their chest. A last-second-attempt was considered valid, if the person had covered more than 50% of the range of motion^11^,^27^.

### Handgrip strength test

To assess handgrip strength, participants performed an isometric handgrip strength test (kg) using a dynamometer. The test was conducted in a sitting position and maximal isometric contraction within 4–5 s was measured (JAMAR compatible handgrip dynamometer adapted to handle different sizes). The better result of two trials (one minute break in between) for each hand was noted^11^,^27^.

### Six minute walking test

The participants had to walk for 6 min as fast and as far as possible. The 6 minute walking test is a valid tool to evaluate aerobic endurance in the elderly. Participants were allowed to slow down and even take a short rest. Every subject performed the test separately without being disturbed by others. They had to walk back and forth on a 30 meter shuttle track and the distance covered within 6 min was registered^11^,^27^.

### Circulating cell-free DNA measurement

Circulating cell-free DNA was measured from frozen EDTA plasma using a QuantiFluor^®^ dsDNA System (Promega), as a plate-based assay. The plasma was thawed and centrifuged for 1 min at 3000 rpm, to remove all insoluble debris. Samples were measured in triplicates, according to the manufacturer’s protocol. To exclude possible interferences from other plasma-derived molecules, each plasma sample was also run without addition of dsDNA dye and used as a blank for the corresponding sample.

### Cytokinesis block micronucleus cytome assay

The cytokinesis block micronucleus cytome (CBMN) assay was performed according to a published protocol^34^. Cells were stimulated to undergo mitosis, using phytohaemagglutinin (PAA, Pasching, Austria) using a concentration of 1 × 10^6 ^cells/mL in culture medium. Samples were incubated at 37 °C and 5% CO_2_. After exactly 44 h, cytochalasin B (Sigma Aldrich, Vienna, Austria) was added to block cytokinesis. After exactly 72 h from the start of the experiment, cells were spotted onto microscope slides, stained (Diff-Qick; Medion Diagnostics, Dudingen, Switzerland) and counted using a bright field microscope (1000-fold magnification; Olympus, Wien, Austria).

Each sample was run in duplicate and two slides of each replicate were produced. From the four resulting slides, 500 cells per slide (2000 per subject) were counted.

To assess chromosomal damage in blood lymphocytes, the frequency of MN, NPBs and NBuds per 2000 binucleated (BN) cells was counted, as well as the number of apoptotic and necrotic cells. Furthermore, the nuclear division index (NDI) was calculated to measure cytostatic effects and the mitogenic response of lymphocytes.

### DNA extraction from whole blood and concentration measurement

DNA was extracted from 100 μl fresh whole blood, using the DNeasy blood and tissue mini kit (Qiagen) according to the manufacturer’s instructions. Extracted DNA was stored at −20 °C. A random subset of samples were measured using NanoDrop 2000c spectrophotometer (Thermo Scientific) and a A260/280 ratio between 1.7 and 1.9 was considered satisfactory. DNA integrity was estimated by agarose gel electrophoresis. DNA concentration for qPCR was measured using the QuantiFluor^®^ dsDNA System (Promega), as a plate-based assay on a FLUOstar OPTIMA microplate reader (BMG Labtech).

### Telomere length measurement by quantitative polimerase chain reaction (qPCR)

Telomere length was measured as described^35^, with modifications. Shortly, SYBR Select Master Mix (Life Technologies) was used to amplify telomeric sequences and single copy gene (36b4). Primers were used at a final concentration of 100 nM. Genomic DNA samples were diluted to a concentration of 2.5 ng/uL, and 2 uL were used in each reaction (5 ng/reaction). 84-base oligonucleotide standards were diluted to a stock solution of 50 pg/uL. To generate a telomere standard curve, 10-fold serial dilutions of the stock solution were prepared. For the single-copy gene (SCG) standard curve, the stock solution was diluted to 0.5 ng/uL, and serial dilutions were prepared thereof. Further details on the standard curve linear ranges are given in the supplementary methods section. All samples and standards were run in triplicate. Assays were run on a QuantStudio™ 6 Flex Real-Time PCR System (Thermo Fisher), using a 384-well block. To account for the effect of inter-plate variability, repeated measures from the same sample were run on the same plate.

Analyses were performed at a manual threshold of 2.461 for both targets, with a qPCR efficiency ranging between 90–110%. Samples with a standard deviation exceeding 0.5 Ct were excluded from the analysis. Absolute telomere length was calculated as previously described^35^. The final results were presented as length in kb of individual telomeres; calculated as telomeric sequence per diploid genome in kb, divided by 92 (number of individual telomeres in the diploid genome).

### Unconjugated bilirubin measurement by high performance liquid chromatography (HPLC)

Unconjugated bilirubin were measured from serum samples, as described previously^12^, using a high-performance liquid chromatograph (Merck, Hitachi, LaChrom, Vienna, Austria) equipped with a photodiode array detector (PDA, Shimadzu,) and a Fortis C18 HPLC column (4.6 × 150 mm, 3 μm) and a phenomenex C18 HPLC guard column (4.0 × 3.0 mm). An isocratic mobile phase consisting of 0.1 M n-dioctylamine in methanol/water (95:5; v/v) and glacial acetic acid was used. Unconjugated bilirubin IX alpha (Frontier Scientific Europe, Carnforth, Lancashire, UK) served as an external standard. Sample and standard preparation and analysis were performed as previously published^12^.

### Biochemical parameters

High sensitive Troponin-T (hs TNT) and NP-were analyzed immediately after blood sampling at a routine laboratory (study lab GmbH, Vienna).

### Oxidized nucleotides in urine

Urine samples were collected on the morning of blood samplings and stored at −20 °C until further analyses. 8-oxo-7.8-dihydro-2′-deoxyguanosine (8oxodG) was measured using an ultra-performance LC and MS/MS^36^. The results were normalized to urinary creatinine concentration determined by the Jaffe reaction.

### Malondialdehyde (MDA)

MDA levels were measured in plasma as described earlier^37^. Shortly, after heating (60 min, 100 °C) plasma samples were neutralized with methanol/NaOH, centrifuged (3 min, 3000 rpm) and MDA was measured using HPLC (excitation: λ = 532 nm, emission: λ = 563 nm, LaChrom Merck Hitachi Chromatography System, Vienna, Austria; HPLC column 125 × 4 mm, 5 μm; Merck, Vienna, Austria).

### Statistical analysis

The sample size calculation was based on a prerequisite alpha level set at 0.05 and a power of >0.85 using G *Power3.1.0^38^ which estimated the sample size to be 86 using isokinetic peak torque (concentric knee extension at an angular velocity of 60°/s.; range of motion 20°–80°) as primary endpoint. Previous studies in a similarly difficult collective show a high drop-out rate of about 35–40%. Therefore, a total of about 120 subjects were included in the study^27^, of which 101 were included in the present analyses at baseline.

For the baseline bivariate correlations we used Pearson and Spearman statistics to test for linear or monotonic relationship. To test for differences between pre- and post-intervention within the same group we used a paired T-test or Wilcoxon signed-rank test. Baseline differences between intervention groups were determined using a one way ANOVA and a Bonferonni correction.

Values from 6-months finishers were paired as pre- and post- intervention and divided by intervention groups. We used a pairwise exclusion. For each group the z-scores were calculated as x − μ_0_/σ_0_, where _x_ is the value, μ_0_ is the group average at baseline and σ_0_ the group standard deviation at baseline. This approach corrects for any inter-group baseline differences. The 6-months difference was calculated from the z-scores for each pair of pre- and post-intervention value.

All analyses were performed using R 3.2.4. or the scipy and numpy^39^ packages for Python 2.7.3. Scatter plots were generated using the matplotlib package for Python 2.7.3^40^, the correlation matrices were generated using the corrplot package for R and the paired line plots using the ggplot2^41^ package for R. PCA and the corresponding figures in supplementary material were generated using the COVAIN toolbox for Matlab^42^.

## Additional Information

**How to cite this article**: Tosevska, A. *et al*. Circulating cell-free DNA, telomere length and bilirubin in the Vienna Active Ageing Study: exploratory analysis of a randomized, controlled trial. *Sci. Rep.* **6**, 38084; doi: 10.1038/srep38084 (2016).

**Publisher's note:** Springer Nature remains neutral with regard to jurisdictional claims in published maps and institutional affiliations.

## Supplementary Material

Supplementary Material

## Figures and Tables

**Table 1:** Baseline Characteristics of 6-months finishers divided by intervention group.

	*RT (n = 23–30)*	*RTS (n = 21–23)*	*CT (n = 20–26)*	*Total (n = 64–79)*
*Age (years)*	82 ± 6	81 ± 8	84 ± 5	83 ± 6
*BMI (kg/m^2^)*	28.97 ± 3.86	29.41 ± 5.31	27.94 ± 5.12	28.76 ± 4.72
*NDI*	1.95 ± 0.08	1.98 ± 0.06	1.98 ± 0.07	1.97 ± 0.07
*MNi frequency/1000 BN cells*	28.60 ± 15.60	28.17 ± 18.43	23.70 ± 9.53	26.90 ± 14.90
*NPBs/1000 BN cells*	1.17 ± 1.21	0.93 ± 0.88	1.36 ± 1.03	1.16 ± 1.06
*NBuds/1000 BN cells*	3.47 ± 2.67	3.35 ± 2.46	4.42 ± 4.85	3.74 ± 3.46
*Stand-up Test (repetitions)*	12.31 ± 3.07	13.77 ± 4.28	12.13 ± 4.44	12.68 ± 3.92
*6 min Walking Test (m)*	384 ± 79	373 ± 99	382 ± 97	380 ± 90
*Body Fat (%)*	33.87 ± 7.36	33.89 ± 9.14	32.59 ± 9.69	33.48 ± 8.55
*Glucose (mg/dl)*	108 ± 25	99 ± 11	107 ± 22	105 ± 21
*hs-Insulin (μIU/ml)*	9.77 ± 6.91	10.43 ± 6.33	8.60 ± 7.74	9.58 ± 6.98
*Cholesterol (mg/dl)*	205 ± 41	209 ± 37	205 ± 38	206 ± 38
*HDL (mg/dl)*	62.57 ± 14.03	66.74 ± 17.88	63.69 ± 20.09	64.15 ± 17.20
*LDL (mg/dl)*	120 ± 37	119 ± 39	118 ± 32	119 ± 35
*Chol/HDL Ratio (%)*	3.40 ± 0.86	3.33 ± 1.01	3.45 ± 0.95	3.40 ± 0.92
*Triglycerides (mg/dl)*	113 ± 38	118 ± 45	114 ± 47	115 ± 43
*Lipoprotein A1 (mg/dl)*	16.22 ± 19.66	36.85 ± 50.70	26.42 ± 37.69	25.58 ± 37.35
*hs-CRP (mg/l)*	6.50 ± 13.27	5.66 ± 9.08	3.96 ± 4.02	5.42 ± 9.75
*UCB (μmol/l)*	8.75 ± 5.68*	6.15 ± 3.33	6.35 ± 2.13	7.16 ± 4.20
*hsTroponin-T (ng/ml)*	0.01 ± 0.01	0.01 ± 0.01	0.02 ± 0.02	0.01 ± 0.02
*NT-pro BNP (pg/ml)*	393 ± 779	280 ± 370	467 ± 672	385 ± 644
*cfDNA (ng/ml)*	185 ± 47	197 ± 55	185 ± 62	188 ± 54
*MDA (μmol/l)*	1.97 ± 0.42	1.89 ± 0.44	1.89 ± 0.36	1.92 ± 0.41
*Telomere Length (kb)*	2.92 ± 1.16	2.76 ± 1.63	3.05 ± 0.94	2.91 ± 1.27

Values are represented as mean ± standard deviation. Differences between intervention groups are marked with *for P-value <0.05, **for P-value <0.01 and ^#^for trend (P < 0.1). One way ANOVA.
