# Supplementary material for: Circulating cell-free DNA, telomere length and bilirubin in the Vienna Active Ageing Study: exploratory analysis of a randomized, controlled trial
Source: Sci Rep. 2016 Dec 1;6:38084. doi: 10.1038/srep38084 (PMC5131485; doi:10.1038/srep38084)
Supplement: Supplementary Material [file srep38084-s1.pdf]

Supplementary material for "Circulating cell-free DNA, telomere length and bilirubin in the Vienna Active Ageing Study: exploratory analysis of a randomized, controlled trial"

Anela Tosevska [1]*, Bernhard Franzke [1], Marlene Hofmann [3], Immina Vierheilig [2], Barbara Schober-Halper [1], Stefan Oesen [1], Oliver Neubauer [1,4], Barbara Wessner [1,3], Karl-Heinz Wagner [1, 2]*

[1] Research Platform Active Ageing, University of Vienna, Althanstrasse 14, 1090, Vienna, Austria

[2] Department of Nutritional Sciences, University of Vienna, Althanstrasse 14, 1090, Vienna, Austria

[3] Centre for Sport Science and University Sports, Department of Sport and Exercise, University of Vienna, Austria.

[4] Queensland University of Technology, Faculty of Health, School of Biomedical Sciences, Institute of Health and Biomedical Innovation (IHBI), Tissue Repair and Regeneration Group, 60 Musk Avenue, Kelvin Grove Campus, Brisbane, QLD 4059, Australia

Supplementary figure S1. PCA clustering of participants in the Vienna Active Ageing Study at baseline. Black circles (1) represent males and blue (2) represent females. There is no clear distinction between the two genders using the 3 most relevant principal components.

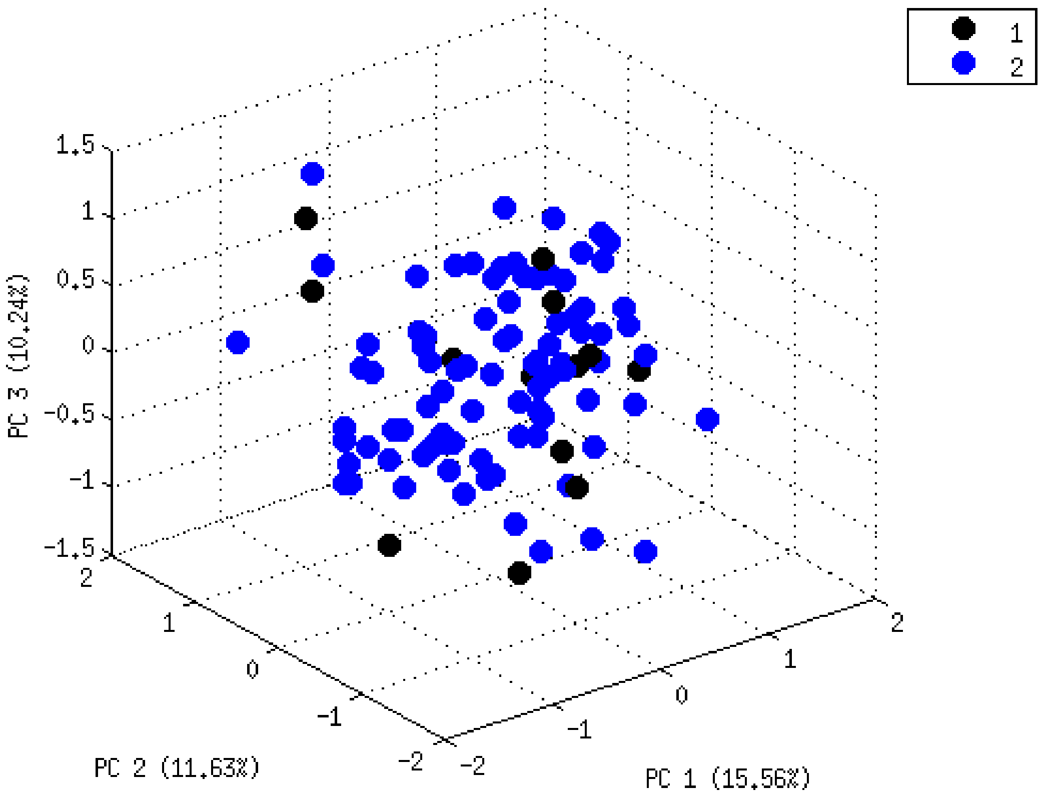

Supplementary figure S2. PCA clustering of participants in the Vienna Active Ageing Study at baseline. Blue circles (1) represent individuals with Gilbert's Syndrome and black (0) represent normobilirubinemic individuals. There is no clear distinction between the two groups using the 3 most relevant principal components.

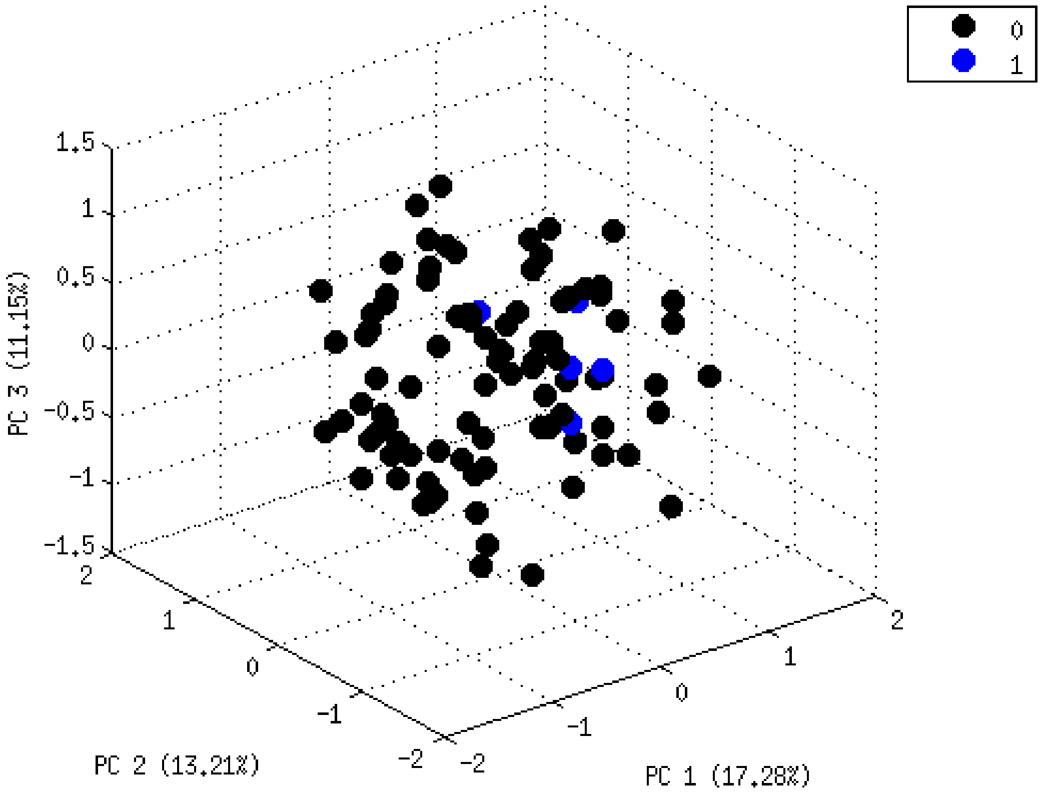

Supplementary figure S3. Histograms representing data distribution of variables at baseline.

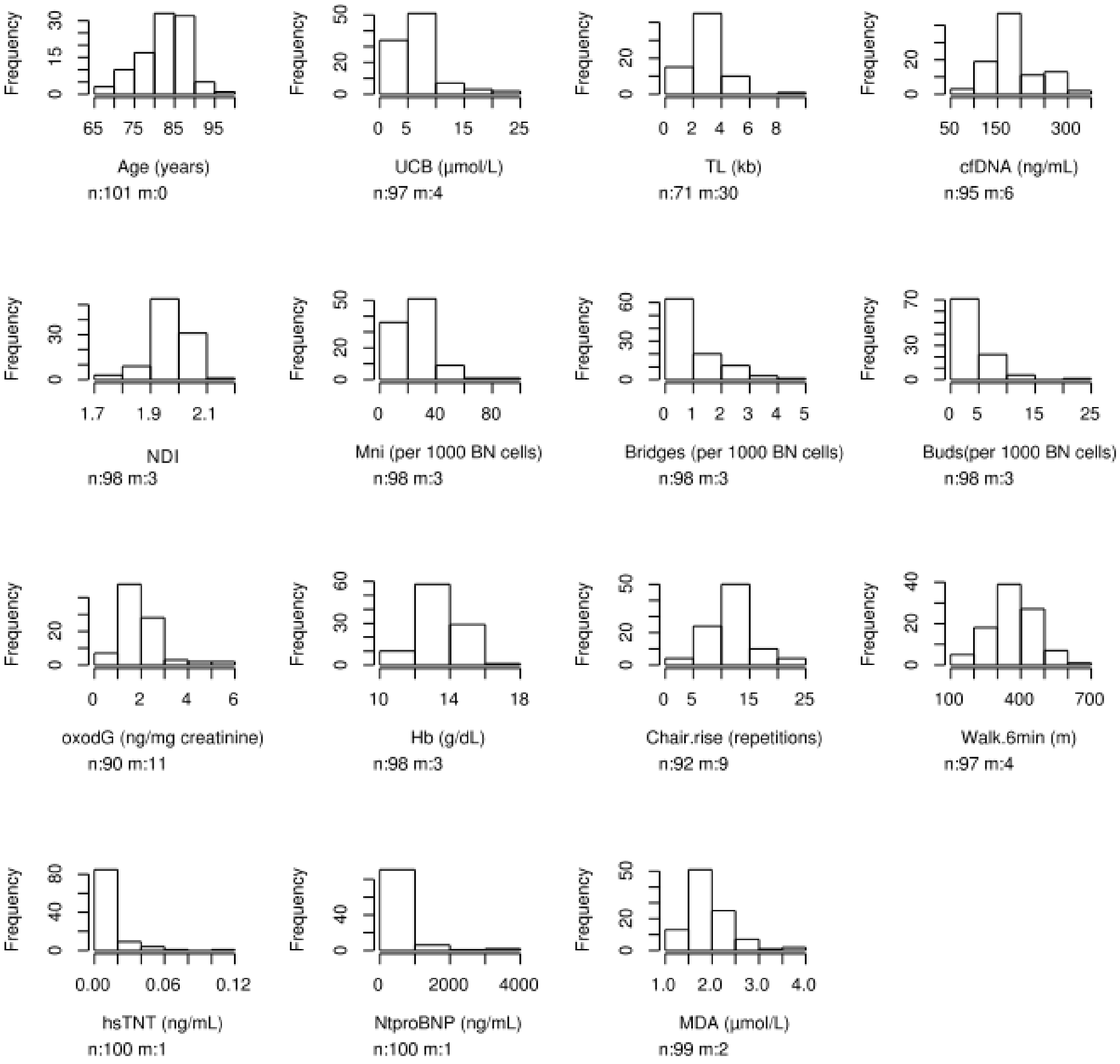

Supplementary figure 4. Scatter plots showing linear correlations at baseline between (a) Telomere length and unconjugated bilirubin, p = 0.051; (b) Unconjugated bilirubin and circulating cell-free DNA, p = 0.014.

a

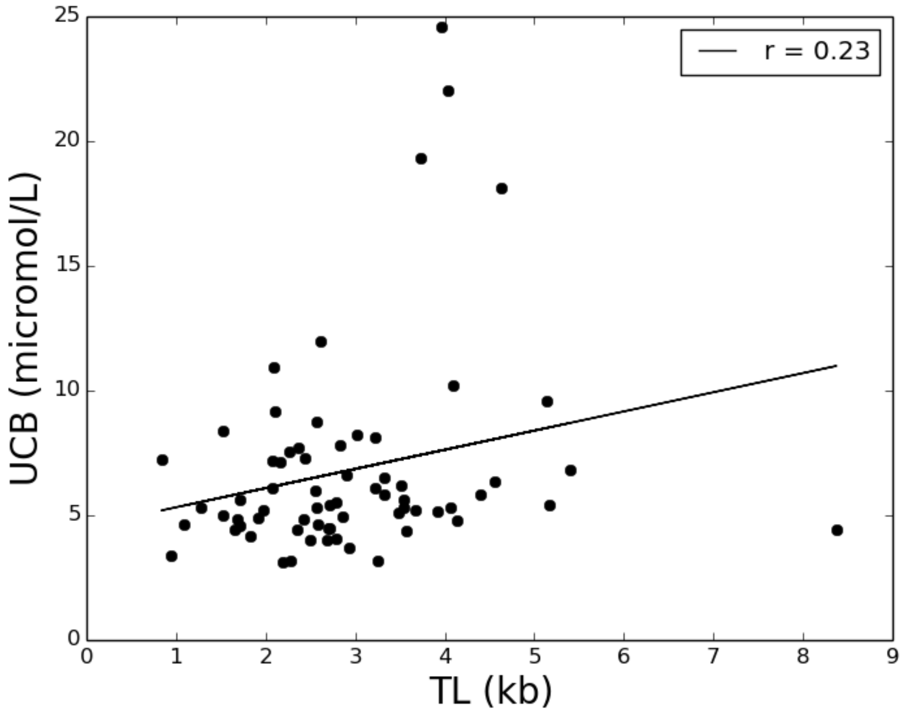

b

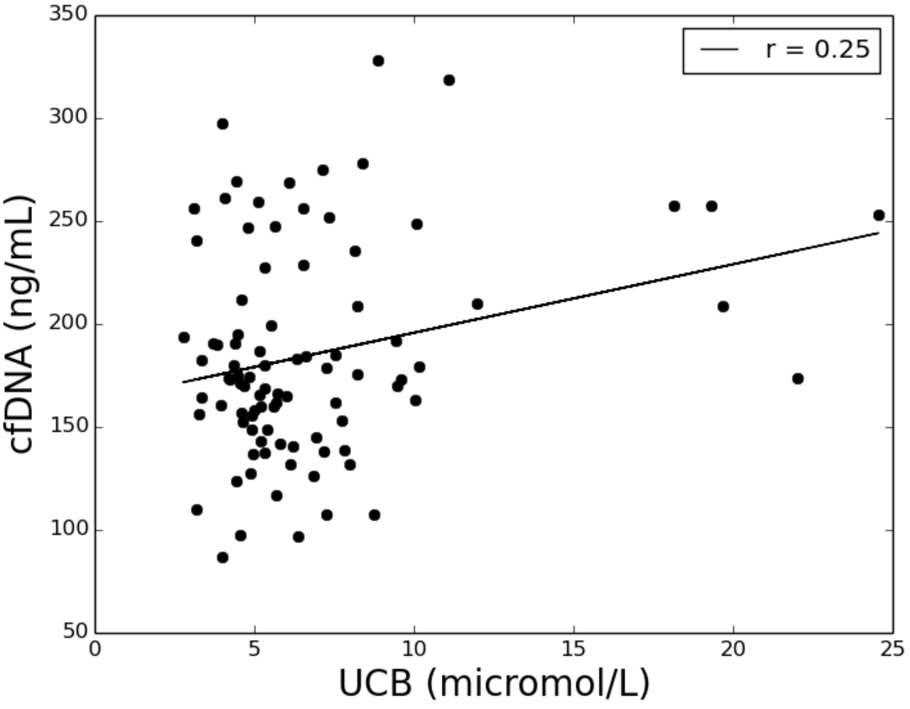

Supplementary methods

Inclusion and exclusion criteria for study subjects

Study inclusion criteria:

- Women and Men aged 65 or more
- Adequate mental condition in order to follow the instructions and to perform the resistance exercise independently (Mini-Mental-State higher than 23)
- Ability to walk 10 meters independently (without orthopaedic devices)
- At least 4 points at the Short Physical Performance Battery (SPPB)

Study exclusion criteria:

- Chronic diseases, which contraindicate a medical training therapy
- Serious cardiovascular diseases (congestive chronic heart failure, severe or symptomatic aortic stenosis, unstable angina pectoris, untreated arterial hypertension, cardiac arrhythmias)
- Diabetic retinopathy
- Manifest osteoporosis
- Anticoagulants (example: Marcomar)
- Regular use of cortisone-containing drugs
- Regular strength training (more than once per week) in the last 6 months before inclusion
- Lack of written declaration of consent for testing physical fitness.

Standard curves and linear ranges of standards used in the qPCR method for absolute telomere length analyses

Here we describe in detail the modifications and validation of the qPCR method for absolute telomere length measurement, according to O'Callaghan and Fenech (2011). The first step was preparation of standard dilutions for telomere standard oligomere (Telo) and single copy gene (36b4). The Telo standards oligomeres were diluted starting from 50 pg/µl to 0.005 pg/µl. Supplementary figure S5 shows a representative standard curve for the telomere standards where the x axes shows the log10 value of the calculated Telo copy number in each standard (see O'Callaghan and Fenech (2011) for detailed step-by-step description on how to calculate the copy number in each standard). All samples fell within the linear range of the standards with Ct values between 24 and 28. The 36b4 oligo standards were diluted starting at 0.5 pg/µl to 5 x 10^{-5} pg/µl. Supplementary figure S6

shows a representative standard curve for the 36b4 standards where the x axes shows the log10 value of the calculated 36b4 copy number in each standard (see O'Callaghan and Fenech (2011) for detailed step-by-step description on how to calculate the copy number in each standard). All samples fell within the linear range of the standards with Ct values between 26 and 28. As seen from figures S5 and S6, the linear range established for this assay differed from the one published previously by (O'Callaghan).

Supplementary figure S5. A representative standard curve for telomere standards. Blue dots represent standards A2-A6; orange squares represent a subset of sample measurement. Over 99% of the samples appeared in the same range.

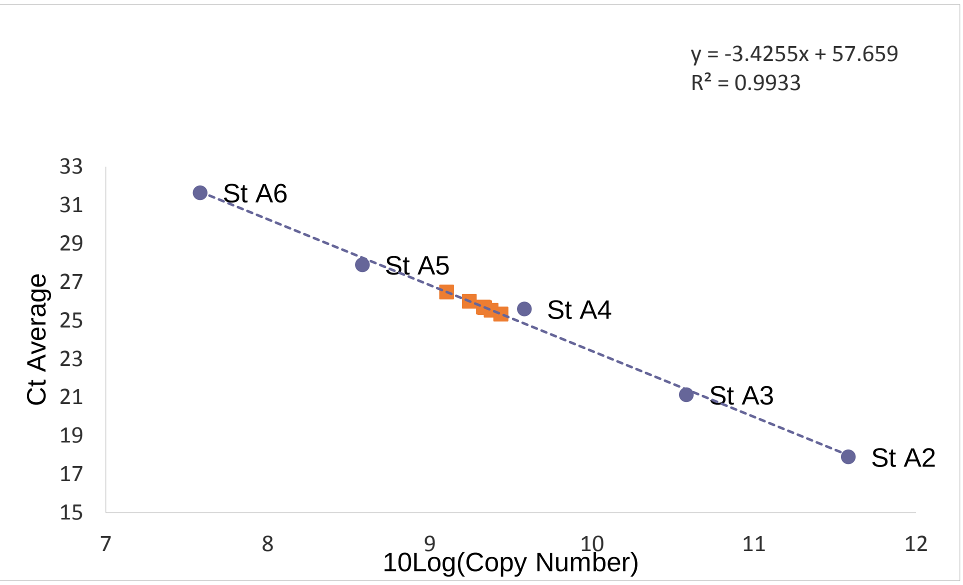

Supplementary figure S6. A representative standard curve for 36b4 standards. Blue dots represent standards B3-B7; orange squares represent a subset of sample measurement. Over 99% of the samples appeared in the same range.

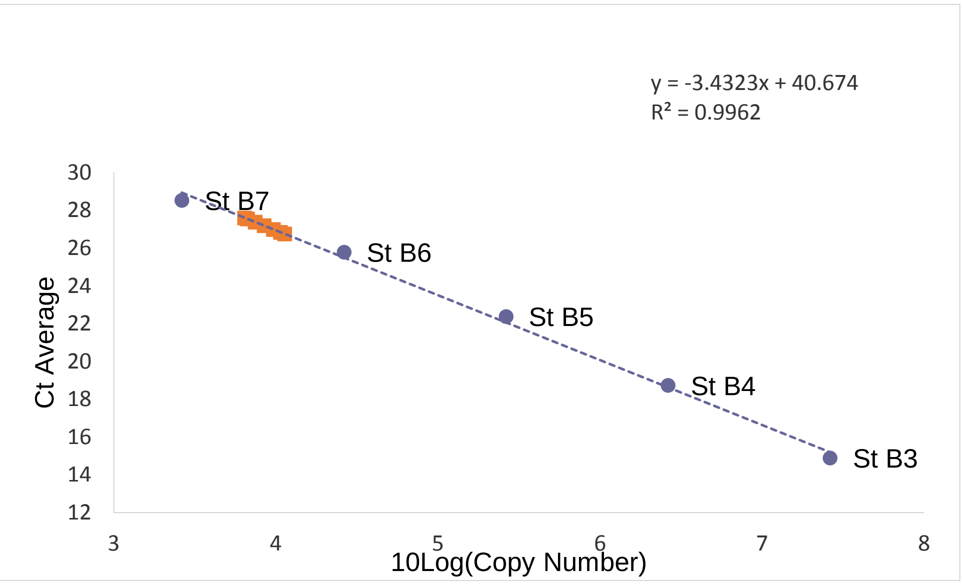

References

O'Callaghan, N. J. and Fenech M. (2011). "A quantitative PCR method for measuring absolute telomere length." Biol Proced Online 13: 3.

	Age	cfDNA	TL	UCB	NDI	Mni	NPBs	Nbuds	Chair.rise	Walk.6min	hsTNT	NtproBNP	Hb	oxodG	MDA
Age	NA	0.593	0.501	0.025	0.952	0.640	0.126	0.201	0.614	0.002	0.033	0.000	0.978	0.761	0.338
cfDNA	0.593	NA	0.545	0.237	0.100	0.787	0.305	0.583	0.994	0.700	0.754	0.911	0.034	0.745	0.353
TL	0.501	0.545	NA	0.047	0.093	0.511	0.000	0.000	0.961	0.361	0.235	0.573	0.852	0.053	0.690
UCB	0.025	0.237	0.047	NA	0.464	0.282	0.043	0.019	0.833	0.194	0.443	0.784	0.013	0.603	0.306
NDI	0.952	0.100	0.093	0.464	NA	0.963	0.003	0.488	0.030	0.013	0.184	0.272	0.296	0.433	0.515
Mni	0.640	0.787	0.511	0.282	0.963	NA	0.534	0.041	0.046	0.104	0.906	0.654	0.726	0.019	0.956
NPBs	0.126	0.305	0.000	0.043	0.003	0.534	NA	0.000	0.046	0.544	0.871	0.103	0.006	0.543	0.001
Nbuds	0.201	0.583	0.000	0.019	0.488	0.041	0.000	NA	0.097	0.194	0.279	0.248	0.008	0.791	0.001
Chair.rise	0.614	0.994	0.961	0.833	0.030	0.046	0.046	0.097	NA	0.000	0.285	0.100	0.010	0.730	0.206
Walk.6min	0.002	0.700	0.361	0.194	0.013	0.104	0.544	0.194	0.000	NA	0.058	0.002	0.149	0.576	0.601
hsTNT	0.033	0.754	0.235	0.443	0.184	0.906	0.871	0.279	0.285	0.058	NA	0.000	0.650	0.970	0.481
NtproBNP	0.000	0.911	0.573	0.784	0.272	0.654	0.103	0.248	0.100	0.002	0.000	NA	0.031	0.124	0.296
Hb	0.978	0.034	0.852	0.013	0.296	0.726	0.006	0.008	0.010	0.149	0.650	0.031	NA	0.083	0.440
oxodG	0.761	0.745	0.053	0.603	0.433	0.019	0.543	0.791	0.730	0.576	0.970	0.124	0.083	NA	0.204
MDA	0.338	0.353	0.690	0.306	0.515	0.956	0.001	0.001	0.206	0.601	0.481	0.296	0.440	0.204	NA

Supplementary table S1. P values for Spearman correlation between variables at baseline. Raw untransformed values were used for the tests.

CONTROL

	cfDNA	TL	UCB	NDI	Mni	NPBs	Nbuds	Chair.rise	Walk.6min	hsTNT	NTproBNP	Hb	oxodG	MDA
cfDNA	NA	0.586	0.887	0.722	0.811	0.822	0.909	0.602	0.014	0.038	0.074	0.639	0.502	0.487
TL	0.586	NA	0.817	0.928	0.636	0.649	0.572	0.591	0.515	0.472	0.721	0.724	0.291	0.726
UCB	0.887	0.817	NA	0.145	0.188	0.063	0.691	0.779	0.443	0.080	0.672	0.002	0.603	0.919
NDI	0.722	0.928	0.145	NA	0.481	0.142	0.027	0.280	0.298	0.000	0.626	0.559	0.882	0.234
Mni	0.811	0.636	0.188	0.481	NA	0.007	0.026	0.631	0.705	0.785	0.419	0.159	0.506	0.498
NPBs	0.822	0.649	0.063	0.142	0.007	NA	0.000	0.852	0.712	0.514	0.225	0.972	0.342	0.350
Nbuds	0.909	0.572	0.691	0.027	0.026	0.000	NA	0.339	0.668	0.067	0.458	0.740	0.864	0.289
Chair.rise	0.602	0.591	0.779	0.280	0.631	0.852	0.339	NA	0.008	0.891	0.595	0.224	0.219	0.346
Walk.6min	0.014	0.515	0.443	0.298	0.705	0.712	0.668	0.008	NA	0.532	0.180	0.850	0.337	0.601
hsTNT	0.038	0.472	0.080	0.000	0.785	0.514	0.067	0.891	0.532	NA	0.077	0.416	0.890	0.865
NTproBNP	0.074	0.721	0.672	0.626	0.419	0.225	0.458	0.595	0.180	0.077	NA	0.027	0.133	0.398
Hb	0.639	0.724	0.002	0.559	0.159	0.972	0.740	0.224	0.850	0.416	0.027	NA	0.099	0.154
oxodG	0.502	0.291	0.603	0.882	0.506	0.342	0.864	0.219	0.337	0.890	0.133	0.099	NA	0.145
MDA	0.487	0.726	0.919	0.234	0.498	0.350	0.289	0.346	0.601	0.865	0.398	0.154	0.145	NA

TRAINING

	cfDNA	TL	UCB	NDI	Mni	NPBs	Nbuds	Chair.rise	Walk.6min	hsTNT	NTproBNP	Hb	oxodG	MDA
cfDNA	NA	0.060	0.039	0.079	0.641	0.342	0.124	0.032	0.924	0.888	0.431	0.110	0.251	0.701
TL	0.060	NA	0.726	0.008	0.680	0.810	0.037	0.011	0.206	0.189	0.392	0.619	0.950	0.914
UCB	0.039	0.726	NA	0.056	0.648	0.021	0.020	0.533	0.339	0.720	0.321	0.198	0.630	0.696
NDI	0.079	0.008	0.056	NA	0.013	0.033	0.000	0.012	0.673	0.347	0.618	0.103	0.901	0.647
Mni	0.641	0.680	0.648	0.013	NA	0.192	0.001	0.394	0.155	0.138	0.574	0.120	0.748	0.774
NPBs	0.342	0.810	0.021	0.033	0.192	NA	0.004	0.631	0.394	0.116	0.204	0.270	0.284	0.149
Nbuds	0.124	0.037	0.020	0.000	0.001	0.004	NA	0.110	0.324	0.170	0.327	0.173	0.980	0.319
Chair.rise	0.032	0.011	0.533	0.012	0.394	0.631	0.110	NA	0.217	0.701	0.401	0.456	0.964	0.248
Walk.6min	0.924	0.206	0.339	0.673	0.155	0.394	0.324	0.217	NA	0.102	0.248	0.159	0.505	0.960
hsTNT	0.888	0.189	0.720	0.347	0.138	0.116	0.170	0.701	0.102	NA	0.361	0.641	0.670	0.255
NTproBNP	0.431	0.392	0.321	0.618	0.574	0.204	0.327	0.401	0.248	0.361	NA	0.530	0.342	0.052
Hb	0.110	0.619	0.198	0.103	0.120	0.270	0.173	0.456	0.159	0.641	0.530	NA	0.477	0.951
oxodG	0.251	0.950	0.630	0.901	0.748	0.284	0.980	0.964	0.505	0.670	0.342	0.477	NA	0.007
MDA	0.701	0.914	0.696	0.647	0.774	0.149	0.319	0.248	0.960	0.255	0.052	0.951	0.007	NA

TRAINING AND SUPPLEMENTATION

	cfDNA	TL	UCB	NDI	Mni	NPBs	Nbuds	Chair.rise	Walk.6min	hsTNT	NTproBNP	Hb	oxodG	MDA
cfDNA	NA	0.874	0.631	0.047	0.596	0.412	0.681	0.048	0.329	0.076	0.791	0.201	0.278	0.450
TL	0.874	NA	0.381	0.422	0.439	0.644	0.439	0.005	0.088	0.935	0.013	0.678	0.639	0.155
UCB	0.631	0.381	NA	0.162	0.467	0.262	0.143	0.642	0.340	0.617	0.790	0.989	0.934	0.385
NDI	0.047	0.422	0.162	NA	0.431	0.038	0.074	0.870	0.789	0.043	0.622	0.708	0.250	0.821
Mni	0.596	0.439	0.467	0.431	NA	0.245	0.147	0.632	0.075	0.869	0.925	0.461	0.521	0.290
NPBs	0.412	0.644	0.262	0.038	0.245	NA	0.019	0.993	0.600	0.617	0.422	0.867	0.238	0.483
Nbuds	0.681	0.439	0.143	0.074	0.147	0.019	NA	0.704	0.667	0.282	0.242	0.097	0.588	0.365
Chair.rise	0.048	0.005	0.642	0.870	0.632	0.993	0.704	NA	0.077	0.981	0.130	0.270	0.947	0.053
Walk.6min	0.329	0.088	0.340	0.789	0.075	0.600	0.667	0.077	NA	0.396	0.682	0.113	0.199	0.258
hsTNT	0.076	0.935	0.617	0.043	0.869	0.617	0.282	0.981	0.396	NA	0.833	0.510	0.003	0.385
NTproBNP	0.791	0.013	0.790	0.622	0.925	0.422	0.242	0.130	0.682	0.833	NA	0.721	0.160	0.167
Hb	0.201	0.678	0.989	0.708	0.461	0.867	0.097	0.270	0.113	0.510	0.721	NA	0.662	0.148
oxodG	0.278	0.639	0.934	0.250	0.521	0.238	0.588	0.947	0.199	0.003	0.160	0.662	NA	0.385
MDA	0.450	0.155	0.385	0.821	0.290	0.483	0.365	0.053	0.258	0.385	0.167	0.148	0.385	NA

Supplementary table S2. P values for Spearman correlation between 6-moths-changes divided by intervention groups. Values were transformed into z-scores according to the baseline mean in for each intervention group and the difference between paired z-scores were used for the tests.
